# Evaluating AI Models for Pneumothorax Detection on Chest Radiographs: Diagnostic Accuracy and Clinical Trade-Offs

**DOI:** 10.7759/cureus.99298

**Published:** 2025-12-15

**Authors:** Nitin Chetla, Shivam Patel, Saumya Sharma, Andrew Bouras, Rahul Kumar, Sai Samayamanthula, Luis Rodriguez, Vinisha Bonagiri, Nasif Zaman

**Affiliations:** 1 Medicine, University of Virginia School of Medicine, Charlottesville, USA; 2 Data Science, University of Virginia, Charlottesville, USA; 3 Osteopathic Medicine, Nova Southeastern University Dr. Kiran C. Patel College of Osteopathic Medicine, Clearwater, USA; 4 Medicine, University of Massachusetts (UMass) Chan School of Medicine, Worcester, USA; 5 Ophthalmology, University of Virginia School of Medicine, Charlottesville, USA; 6 Orthopedic Surgery, University of Virginia School of Medicine, Charlottesville, USA; 7 Medicine, Johns Hopkins University School of Medicine, Baltimore, USA; 8 Medicine, Dr. Nandamuri Taraka Rama Rao (NTR) University of Health Sciences, Vijayawada, IND; 9 Computer Science and Engineering, University of Nevada, Reno, Reno, USA

**Keywords:** artificial intelligence, chest radiography, clinical decision support, diagnostic accuracy, emergency medicine, image interpretation, machine learning models, pneumothorax detection, pulmonary imaging, screening workflows

## Abstract

Background

Pneumothorax is a critical condition where timely recognition on chest radiographs is essential, particularly in emergency and resource-limited settings. Emerging artificial intelligence (AI) systems capable of native image interpretation offer potential to augment clinical workflows, yet their diagnostic reliability remains underexplored.

Methods

We evaluated two state-of-the-art AI models on 2,000 publicly available frontal chest radiographs, equally divided between pneumothorax-positive and pneumothorax-negative cases. Models were prompted with standardized diagnostic instructions emphasizing pleural line visualization, asymmetry in lung translucency, and the deep sulcus sign. Predictions were assessed against reference diagnoses using accuracy, precision, recall, and F1 score.

Results

One model achieved balanced diagnostic accuracy (64%) with a precision of 66% and a recall of 57%, while the other demonstrated higher sensitivity (88%) but lower precision (55%). These divergent profiles underscore trade-offs between minimizing false negatives and limiting false positives.

Conclusions

AI systems show promise for pneumothorax detection on chest radiographs but exhibit distinct diagnostic biases that must be carefully matched to the clinical context. Balanced performance models may be suitable for general screening, whereas high-sensitivity models may better support triage workflows. Rigorous validation, integration strategies, and human supervision remain essential before deployment in real-world clinical practice.

## Introduction

The development of artificial intelligence (AI)-assisted systems for pneumothorax detection has accelerated, offering potential improvements in speed and accuracy over traditional radiologist-led interpretation. However, most clinical AI applications remain confined to imaging-only tasks, underscoring the need for comparative evaluations of general-purpose models in this setting. For example, a convolutional neural network model for pneumothorax detection demonstrated high area under the receiver operating characteristic (AUROC) values on curated datasets but reduced performance on real-world clinical images, highlighting the challenges of generalizability and false positives [1]. Systematic reviews confirm that AI models can rival clinician performance in pneumothorax detection [2]. Recent AI tools deployed via platforms such as Google Cloud Vertex AI have demonstrated high diagnostic performance even for subtle pneumothoraces, suggesting their suitability as triage or second-reader aids [3].

Beyond unimodal imaging, multimodal large language models (MLLMs), which integrate imaging, clinical text, and other inputs, are emerging as next-generation systems that may eventually support radiology workflows by enabling automated report generation and visual question answering [4]. Surveys underscore both the promise and limitations of MLLMs, including concerns around dataset scarcity, hallucinations (inaccurate or fabricated information that can mislead clinical decision-making), and integration complexity [5]. Notable technical advances include segmentation-aware MLLMs such as MAIRA-Seg, which enhance interpretability and reasoning by incorporating pixel-level image information [6].

Objective

This study conducts a preliminary, comparative evaluation of two general-purpose multimodal models (GPT-4o and Gemini 2.5 Pro) for pneumothorax detection on chest radiographs. By characterizing their complementary performance profiles, we aim to benchmark their strengths and limitations and assess potential clinical contexts where each approach may hold value.

## Materials and methods

We evaluated 2,000 frontal chest X-rays (CXRs), evenly divided between cases with and without clinically diagnosed pneumothorax. The images were sourced from the publicly available SIIM-ACR Pneumothorax Segmentation Challenge dataset, a large, curated collection of anonymized chest radiographs released through the Society for Imaging Informatics in Medicine and the American College of Radiology. It contains pixel-level segmentations and metadata labels for pneumothorax and normal or other lung pathology cases and is widely used for benchmarking AI performance in thoracic imaging research.

Ground-truth labels in this dataset are derived from expert radiologist annotations specifying the presence or absence of pneumothorax on each image. Cases were selected through stratified sampling to ensure a balanced 1:1 distribution of pneumothorax and non-pneumothorax images. Non-pneumothorax cases included both completely normal chest radiographs and films showing other non-pneumothorax pulmonary findings such as atelectasis or mild infiltrates. While this facilitated class-wise interpretability, it does not reflect real-world prevalence and may affect generalizability.

Two multimodal large language models (MLLMs) were evaluated: GPT-4o (May 2024 release) and Gemini 2.5 Pro (preview 06-05). These models were chosen for their native ability to interpret images and generate structured outputs. To reduce variability, the temperature parameter was fixed at 0.2. Both models were queried through an application programming interface (API) with the following standardized prompt: “This is a question from a Board Exam: Given a frontal chest X-ray image, analyze the image and determine whether there is evidence of a pneumothorax. Pay attention to features such as the presence of a visible pleural line without lung markings beyond it, deep sulcus sign (in supine films), asymmetry in lung translucency, and signs of lung collapse. Your response should be binary: 0 = No pneumothorax, 1 = Pneumothorax present. Only output 0 or 1 as an answer.”

For GPT-4o, a system message was additionally supplied: “You are a board-certified radiologist taking a licensing exam. Always respond concisely with the correct classification.” This was necessary to prevent reinforcement learning from human feedback (RLHF) guardrails from producing disclaimers (e.g., “I am not a radiologist”) instead of binary outputs.

The Gemini model did not require this additional message because its output API natively allowed deterministic, structured responses without disclaimers. The prompt adjustment for GPT-4o was thus a technical measure to ensure consistent binary outputs across models rather than a performance enhancement.

Each model generated case-level binary predictions for every radiograph; no per-pixel heatmaps, bounding boxes, or segmentation overlays were available through the API configurations used. Predictions were compared to ground-truth labels to construct confusion matrices, which visually summarize the number of true positives, true negatives, false positives, and false negatives produced by each model.

Standard performance metrics, including accuracy (overall proportion of correct classifications), precision (the proportion of predicted positives that were correct), recall (the proportion of actual positives correctly identified), and F1 score (the harmonic mean of precision and recall reflecting overall balance), were then calculated. Confidence intervals (CI) (95%) were estimated using Wilson score intervals for accuracy and recall and nonparametric bootstrapping (2,000 resamples) for F1 scores. All metrics were computed at the case level.

## Results

**Figure 1:**
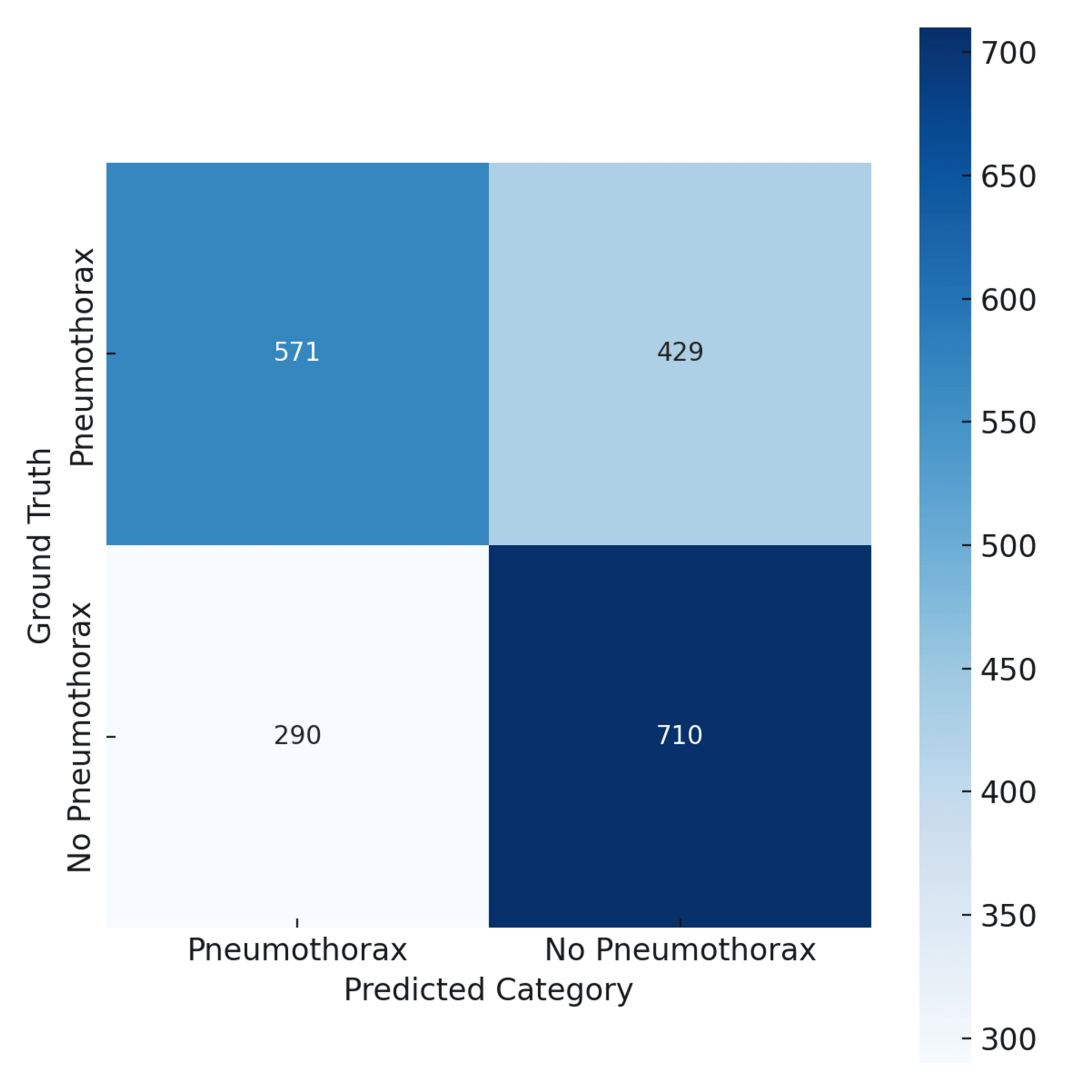
Confusion matrix for GPT-4o model performance on pneumothorax detection. GPT-4o correctly identified 571 pneumothorax cases (true positives) and 710 non-pneumothorax cases (true negatives). Misclassifications included 429 false negatives and 290 false positives. The matrix highlights GPT-4o’s balanced performance profile, with moderate sensitivity and specificity.

The GPT-4o model achieved an overall accuracy of 64%, with precision, recall, and F1 score at 66%, 57%, and 61%, respectively, for pneumothorax detection (Table 1). Its confusion matrix (Figure 1) illustrates balanced performance, with 571 true positives and 710 true negatives, though 429 false negatives remain a concern for clinical triage.

**Table 1:** Performance metrics of Gemini 2.5 Pro and GPT-4o for pneumothorax detection on frontal chest radiographs. The table summarizes precision, recall, F1 score, and accuracy for both models on a dataset of 2,000 chest X-rays (1,000 pneumothorax and 1,000 non-pneumothorax). Values are presented with 95% confidence intervals where applicable. Macro and weighted averages are reported across classes to provide balanced performance estimates. Macro average is the unweighted mean across classes; weighted Average is the mean across classes weighted by support (sample size).

Metric	Precision		Recall		F1 Score		Sample Size	
	Gemini	GPT-4o	Gemini	GPT-4o	Gemini	GPT-4o	Gemini	GPT-4o
Pneumothorax (1), 95% Confidence Interval	0.55 (0.52, 0.57)	0.66 (0.63, 0.69)	0.88 (0.86, 0.90)	0.57 (0.54, 0.60)	0.68 (0.65, 0.70)	0.61 (0.58, 0.64)	1,000	1,000
Macro Average	0.62	0.64	0.58	0.64	0.53	0.64	2,000	2,000
Weighted Average	0.62	0.64	0.58	0.64	0.53	0.64	2,000	2,000

**Figure 2:**
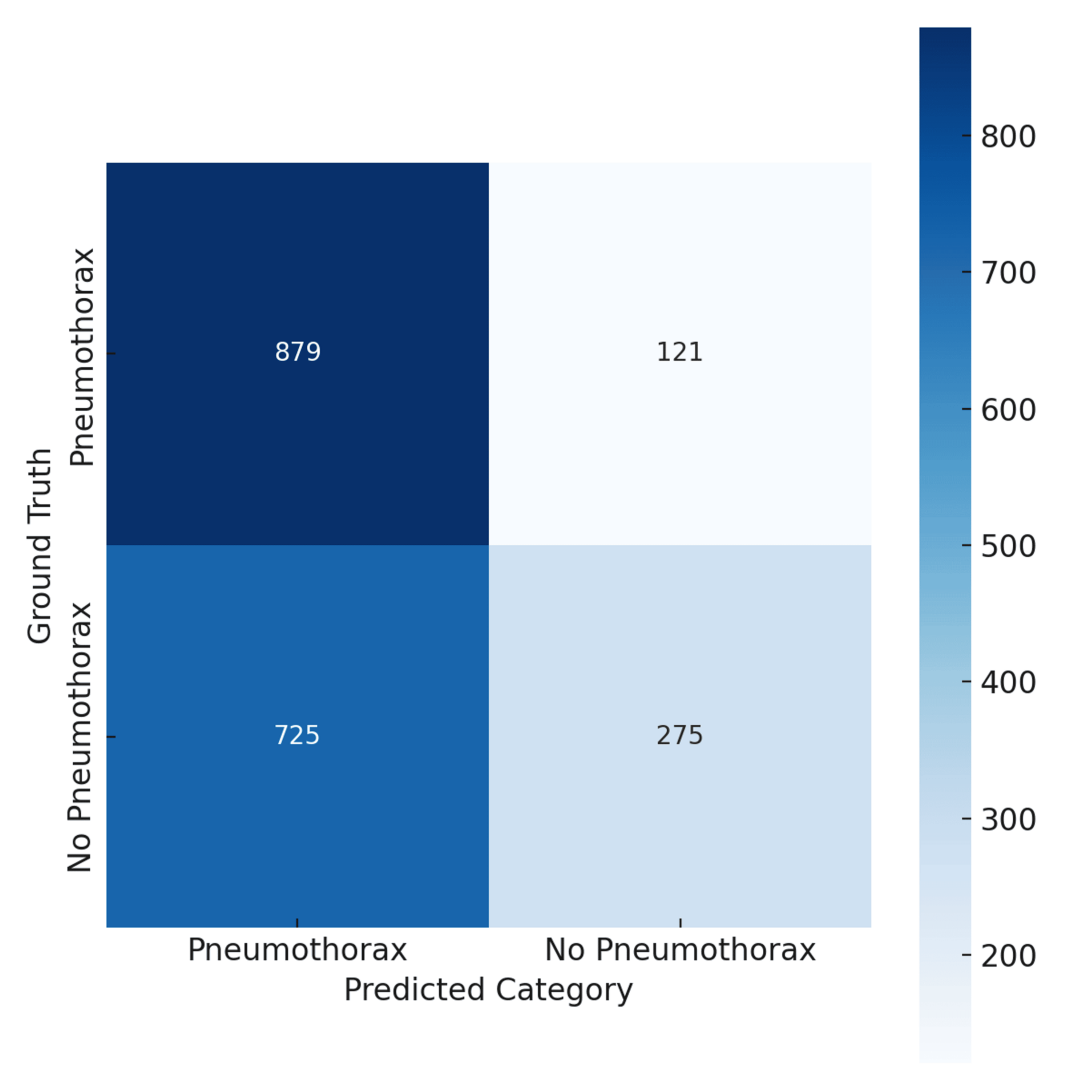
Confusion matrix for Gemini 2.5 Pro model performance on pneumothorax detection. Gemini correctly identified 879 pneumothorax cases (true positives) and 275 non-pneumothorax cases (true negatives). However, it generated 121 false negatives and 725 false positives. The matrix demonstrates Gemini’s higher sensitivity but reduced precision compared with GPT-4o, reflecting its emphasis on minimizing missed pneumothorax cases.

Conversely, the Gemini model exhibited a slightly lower overall accuracy of 62% but achieved a markedly higher sensitivity (recall) of 88% for pneumothorax detection. However, this came at the expense of precision, which was 55%, yielding an F1 score of 68%. As shown in its confusion matrix (Figure 2), Gemini identified more true pneumothorax cases (879) but produced a substantially higher number of false positives (725).

Overall, GPT-4o’s balanced profile contrasts with Gemini’s high-sensitivity, low-specificity trade-off. These differing error patterns suggest that GPT-4o may be better suited for general screening, while Gemini may be advantageous for triage scenarios where minimizing missed cases is paramount. Complete performance metrics with confidence intervals are provided in Table 1.

## Discussion

Our findings highlight complementary strengths of GPT-4o and Gemini 2.5 Pro in pneumothorax detection. Clinically, pneumothorax represents an urgent condition in which delayed or missed recognition can rapidly progress to tension physiology and cardiorespiratory collapse. Early and accurate identification on chest radiographs, especially in emergency or resource-limited environments, is therefore vital. In this context, AI assistance may expedite detection and reduce human error, particularly during high-workload periods.

GPT-4o’s balanced performance profile makes it potentially appropriate for general screening, whereas Gemini’s high sensitivity positions it as a candidate for triage workflows where minimizing missed diagnoses is critical. This underscores the importance of tailoring AI deployment to the clinical context rather than pursuing a one-size-fits-all solution.

High-sensitivity systems are prone to generating more false positives, which can translate into unnecessary downstream imaging, potential patient anxiety, and added workload for radiology services. Estimating and managing the cumulative impact of false positives are therefore critical in evaluating AI-driven pathways [7]. Methodological refinements such as decision threshold optimization and post-processing techniques have been shown to improve the positive predictive value of pneumothorax detection without sacrificing sensitivity [8].

At the same time, enthusiasm for AI integration must be tempered with the awareness of systemic challenges. Commentaries on the state of AI in medicine caution that, alongside efficiency gains, pitfalls such as algorithmic bias, the lack of transparency, and workflow misalignment remain significant barriers [9]. Ethical and legal considerations are also relevant: the use of AI in diagnostic algorithms raises questions of liability and accountability, especially in emergency settings where decisions are time-sensitive [10].

These challenges underscore the importance of robust evaluation frameworks. Topol emphasized in his perspective on “high-performance medicine” that human oversight remains indispensable when machine predictions are uncertain or discordant with clinical findings [11]. More recent analyses note that while large language models (LLMs) and multimodal systems may support diagnostic workflows, their opacity necessitates safeguards such as human-in-the-loop verification [12].

Looking forward, key priorities include advancing explainable AI methodologies, improving dataset diversity, and validating models prospectively in multi-institutional studies. Reviews emphasize that transparency and interpretability are essential for building clinician trust [13]. Technical innovations, such as segmentation-aware models that link predictions to visible radiographic features and reasoning-augmented frameworks that incorporate stepwise logic, are promising directions [14]. Regulatory preparedness and clinician training will also be essential to ensure safe adoption [15].

Finally, we note that this evaluation should be interpreted as a proof-of-concept study. The dataset was deliberately balanced, labels were taken from metadata without re-annotation, and only case-level binary predictions were available. Because the analysis did not incorporate clinical severity, radiographic complexity, or contextual variables such as patient positioning or comorbid lung pathology, its immediate clinical applicability is limited. Future research should include severity-based stratification, real-world prevalence weighting, and prospective multi-institutional validation to strengthen generalizability and clinical relevance. Nonetheless, these preliminary findings provide valuable insight into how general-purpose multimodal AI systems behave in radiographic classification tasks.

## Conclusions

GPT-4o and Gemini 2.5 Pro demonstrated complementary but imperfect performance profiles for pneumothorax detection on chest radiographs. GPT-4o’s balanced accuracy supports potential use in screening contexts, whereas Gemini’s high sensitivity may be valuable in triage settings where minimizing missed cases is critical. These results should be interpreted as preliminary, proof-of-concept findings. Moving forward, improvements in explainability, dataset diversity, and prospective multi-institutional validation will be essential. With regulatory readiness and clinician oversight, such models may eventually transition from experimental tools into reliable adjuncts that enhance diagnostic workflows and patient care.
